# Effect of fiber orientation on the mechanical properties of multi layers laminate nanocomposites

**DOI:** 10.1016/j.heliyon.2020.e03167

**Published:** 2020-01-15

**Authors:** Amal Nassar, Eman Nassar

**Affiliations:** Higher Technological Institute, Mechanical Engineering Department, Tenth of Ramadan City, Egypt

**Keywords:** Materials science, Nanotechnology, Nanomaterials, Nanocomposites, Microstructure, Mechanical properties, Thermal properties, Barcol hardness, Fiberglass, Nano filler

## Abstract

The effect of fiberglass type and adding a very small amount of Nano filler in the resin on mechanical and thermal properties of multilayers laminate composite has been studied. The results show clearly that laminate composites can be achieved by controlling the fiberglass type and by dispersing nanoparticles in the resin. Using continues fiber glass helps to increase the impact strength by 17 %–24% compared with samples with random fiberglass. The barcol hardness of continues fiberglass composite is 7% higher than random fiberglass composite. The results of this study show that using small amount form Nano filler in the resin could produce a laminate composite with excellent thermal and mechanical properties.

## 1 Introduction

The composite material is a combination of two different materials. The combination of laminated sheets of fabric materials and resin is called laminate composite. In order to increase the mechanical properties of the composites, different techniques can be used such as adding hard materials (ceramics, glass mats or silicone), using chemical additives or making coating layer [1]. Fiberglass is one of the promising material that can be used in laminate composite production. It is made by heating glass until it is molten when it is formed to very thin fibers by forcing it through superfine holes [2]. Adding resins to fiberglass give it the required strength to be used in producing different common items such as doors, sporting equipment, and swimming pools [3]. Fiber content effect on the composite properties, increasing in fiber leads to an increase in tensile properties [4, 5]. The processing techniques also affect the composite properties [6].

Hybrid composite refers to the matrix reinforced with more than two fillers [7]. The benefits of using hybrid composites depend on the fact that the characteristic of single filler can be enhanced by another filler [8]. There are many nano fillers used today such as carbon nanotube, nano clay and nano carbon fibers [9]. The Graphene Nano Platelet GnP is widely nano filler in many advanced applications such as batteries, sensors and super capacitors [10, 11]. The GnP transfers the stress efficiently improves the capability of load carrying that is due to large specific surface area. Adding GnP nano filler to polymeric composite leades to improve dynamic, thermal, electrical and mechanical properties of the composites [12]. There are many reserches reported that graphene nano composites showed stiffness and strength higher than carbon nano composites and with low cost [9, 13].

Thermal and dynamic behavior of polymers are important parameters in mechanical design, Dynamic Mechanical Analysis DMA is a widely used technique to determine viscoelastic and morphology of polymer and composite materials [14]. Polyurethane resin reinforced with fiberglass (FRP) is used to produce wall panels, that are used in washable low-maintenance walls. Polyurethane resin is a thermosetting polymer that formed to final shape by mixing it with a catalyst such as methyl ethyl ketone or benzoyl peroxide [15]. In the current study, different combinations (according to fiberglass type and wt% from Graphene nano platelets GnP) were examined to find out the properties of each combination. Tensile tests were used to study the tensile behavior of the laminate Composite. Dynamic analysis tests were conducted on the DMA machine to evaluate the dynamic behavior of composite. Several works [16, 17] reported that adding nano fibers or particles could improve composites properties such as thermal and dynamic properties. Moreover, the nano filler plays a great role in the relaxation of chains macromolecular polymeric [18, 19]. The primary focus in current research is study the effect of fiberglass type and the effect of adding very small amount of Nanofiller to resin on mechanical and thermal properties of multilayers laminate composite.

## 2 Experimental work

### 2.1 Materials

**Figure 1:**
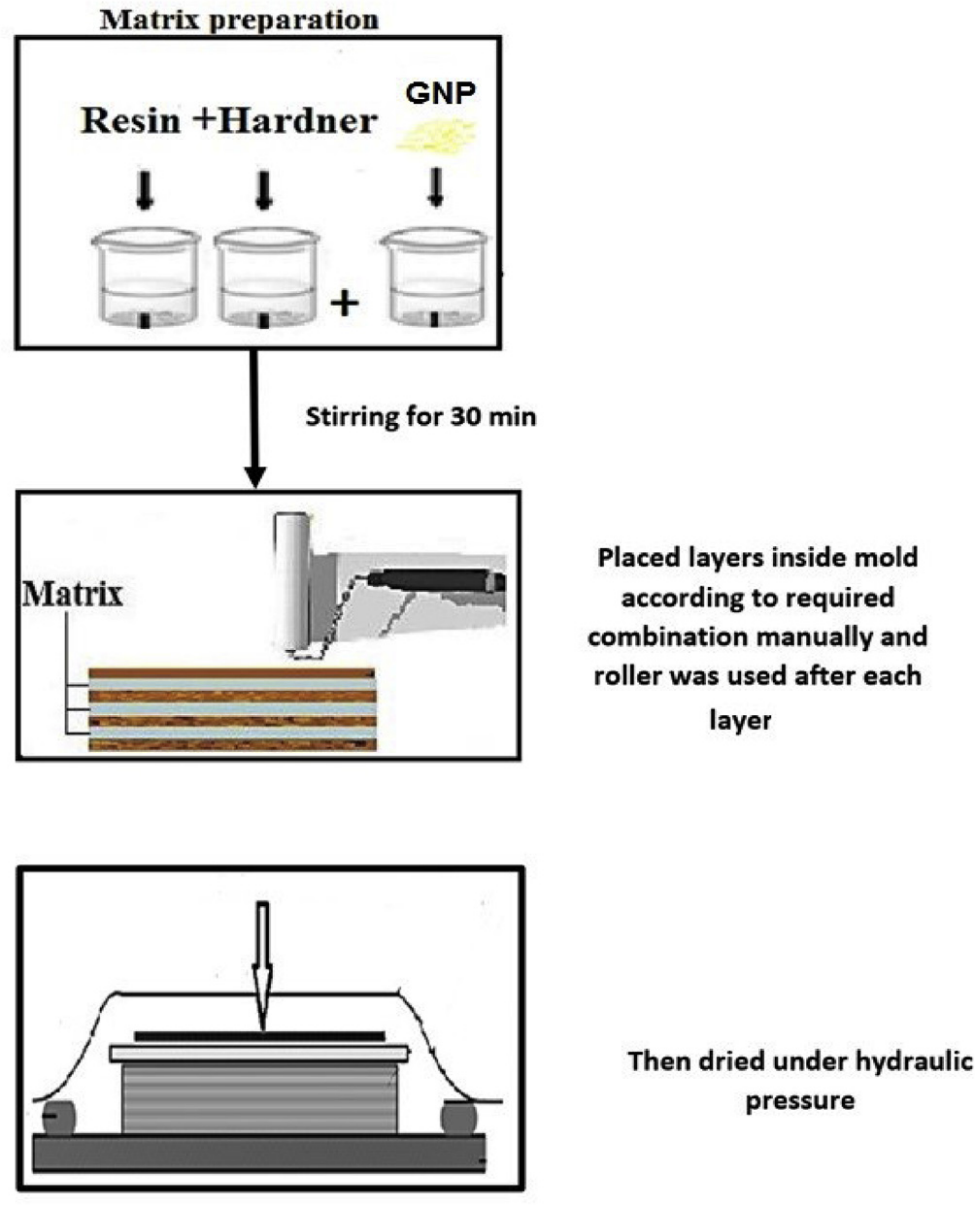
Preparation steps used in the investigation.

The material used in this study consists of fiberglass with continuous fibers (woven roving) and with random fibers obtained from Fibrex Egypt. Fabric weights of continuous and random fiber were 345 g/m^2^ and 363 g/m^2^ respectively. The average length of random fibers used in the investigation was 1.7 mm. Vacuum casting Polyurethane resin (PX 225) and acid anhydride hardener were purchased from Axson Middle East, Table 1 shows physical properties of the selected resin at room temperature. The ratio of resin to hardener was 100 to 75 by wt %. In order to remove any existing moisture in fiberglass, it is placed in an oven for 18 h at 80 °C. The specimens were made manual by placed layers inside aluminum mold (A6061) according to required combination to produce a square panel (20 × 20 cm^2^), then the panel was cured under hydraulic pressure of 50 kg/cm^2^) for 50 min at room temperature (23 °C ±1). In this work, layer from liquid wax is applied on the mold to facilitate the panel removal, Figure 1 shows preparation steps used in the investigation. Each panel has 12 layers of fibers with an approximate thickness equal to 3 mm. The details of the combination of the fibers are shown in Table 2. In order to prepare the random fiber layer digital balance was used to weight the required amount from random fiber to reach the required volume fraction of glass fiber. The panels were cut into samples according to the required geometry by laser cutting. The resulting samples were cured at 90 °C for 2 h to finish the crosslinking reaction.

**Table 1:** Physical, mechanical and thermal properties of the polyurethane resins.

Properties	Value
Flexural modulus (MPa) obtained using ISO 14130 (Instron, Hungary)	2500 (MPa)
Viscosity at 25°C (mPa.s) obtained using A21 Digital Brookfield rotational viscometer ASTM D4402, (Xian Zealchon Electronic Technology Co., Ltd., China)	4
Density at 25°C (g/cm^3^) obtained using liquid densimeter DA-300W, (Dongguan Hongtuo Instrument, china)	1.17
Hardness (Shore D) obtained using Shore Hardness Meter (Shore Durometer, China)	80

**Table 2:** GnP wt% in each sample.

Sample no.	Type of glass fiber	GnP (wt%)
1	Continuous	0
2	Random	0
3	Continuous	0.1
4	Random	0.1
5	Continuous	0.3
6	Random	0.3
7	Continuous	0.4
8	Random	0.4
9	Continuous	0.5
10	Random	0.5

The volume fraction of fiber in the composite is an important parameter and it is calculated according to the following equation [20]:

(1) $$Vf=\frac{Mf}{\rho f}$$

where, Vf= volume of glass fiber, Mf= Mass of glass fiber, ρf = Density of glass fiber

Fiber used is fiberglass has density $\rho f=1.8\;g/cm^{3}$

Resin used has density: = 1.17 g/cm^3^

By measuring the weight of fiber and dividing it with its density, Vf= 41 cm^3^

By measuring the weight of matrix (resin + hardener) and dividing it with its density, Vm= 96 cm^3^

(2) Vc= Vf +V

Vc= 41+96=137 cm^3^

The volume fraction of fiber =$\frac{Vf}{Vc}$ = $\frac{41}{137}$= 0.299 ≈ 0.3

The volume fraction of matrix =$\frac{Vm}{Vc}$ = $\frac{96}{137}$= 0.7

**Figure 2:**
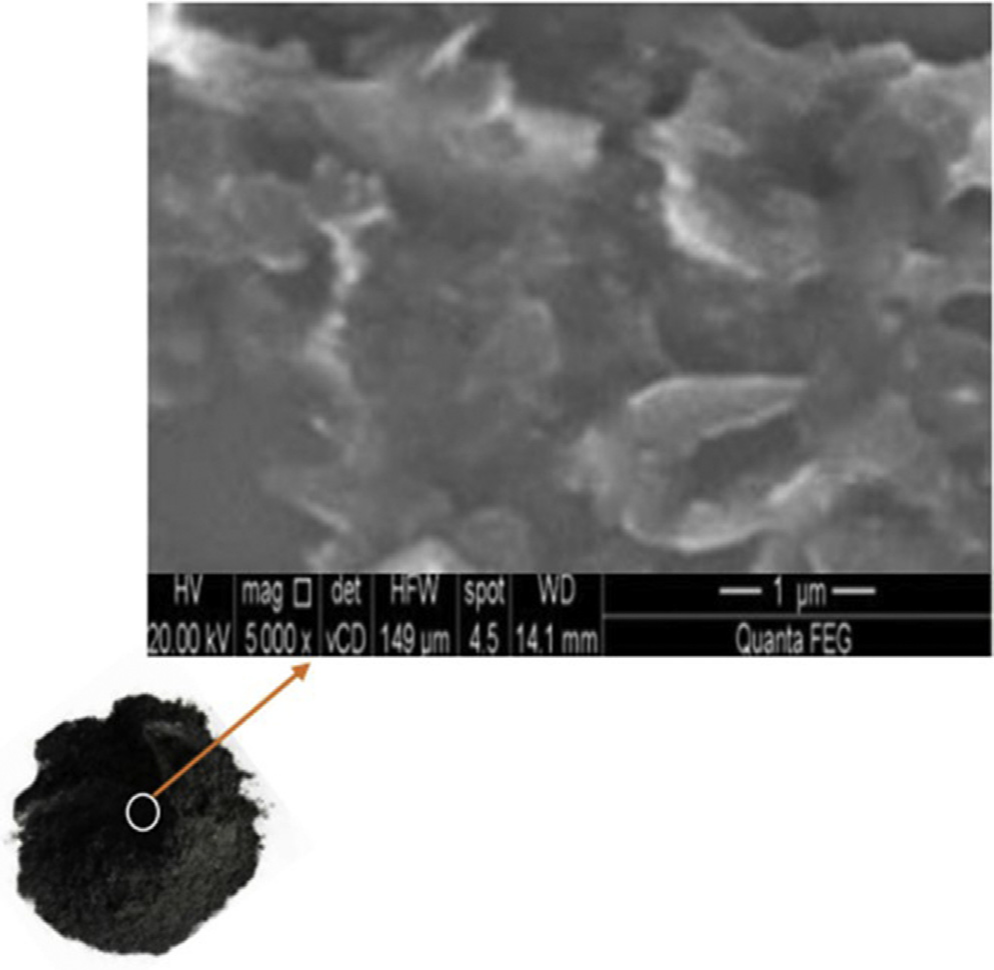
SEM image of Graphene Nanoplatelets.

Graphene Nanoplatelets (GnP grade: Nano19) were purchased from Asbury, Inc in a powder form used as reinforcement. Graphene is a 2D sheet of sp2 bonded carbon atoms, organized in a hexagonal lattice [21]. GnP has unique morphology and size which enhanced its mechanical and thermal properties [22]. The orientation of GnP was randomly distributed in the matrix, Figure 2 shows an SEM image of Graphene Nanoplatelets. The percentage of GnP for each combination were (0.1,0.3, 0.4 and 0.5%). The densities of samples were calculated according to Archimedes principle shown below:

(3) $$\text{Density (}\rho \text{)}=\frac{\text{ρ of fluid }\times \text{weight in air}}{\text{weight in air}-\text{ weight in fluid}}$$

### 2.2 Mechanical properties

**Figure 3:**
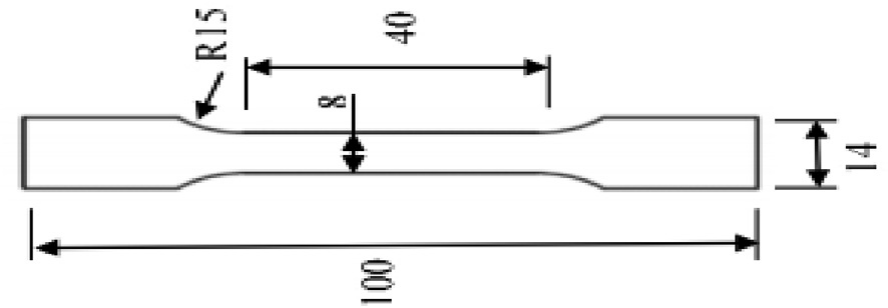
Geometry and dimensions of the tensile test sample.

Barcol hardness values were measured to study the effect of wt% of Nanofiller and fiberglass of type on the composite hardness. Hardness tests are conducted according to (D2583-87 ASTM). Tensile tests were used to study how the laminate composite break or deform as a function ofapplied load at room temperature by using a Computer Control Polymer Universal Testing machine/universal testing machine UTES-20 (FIE, India). The dimensions of the tensile sample are illustrated in Figure 3 based according ASTM E8 . Surfaces of fracture in some samples were gold sputtered and then observed with a scanning electron microscope to study the failure mechanisms. Samples are examined directly by scanning electron microscope JSM 6480 LV (JEOL, USA) at 30 Kv. Charpy impact test was used to study the impact of absorbing properties for the composites. Tests were obtained using Charpy Impact Testing Machine (XJJD Electronic, China) for the rectangular shape samples with 14 mm as a length, and 30 mm as a width and 60 mm thickness mm and V-notch angle equal 60°.

### 2.3 Thermal properties

Thermal analysis of samples was conducted using DSC Analysis of the graft copolymers samples was conducted using differential scanning calorimetric analyzer (DSC NETZSCH 214). The measurement was carried out under N2 gas with a scanning rate of 10 °C/min and temperatures ranged from 30-250 °C.

## 3 Results and discussion

### 3.1 Microstructure

**Figure 4:**
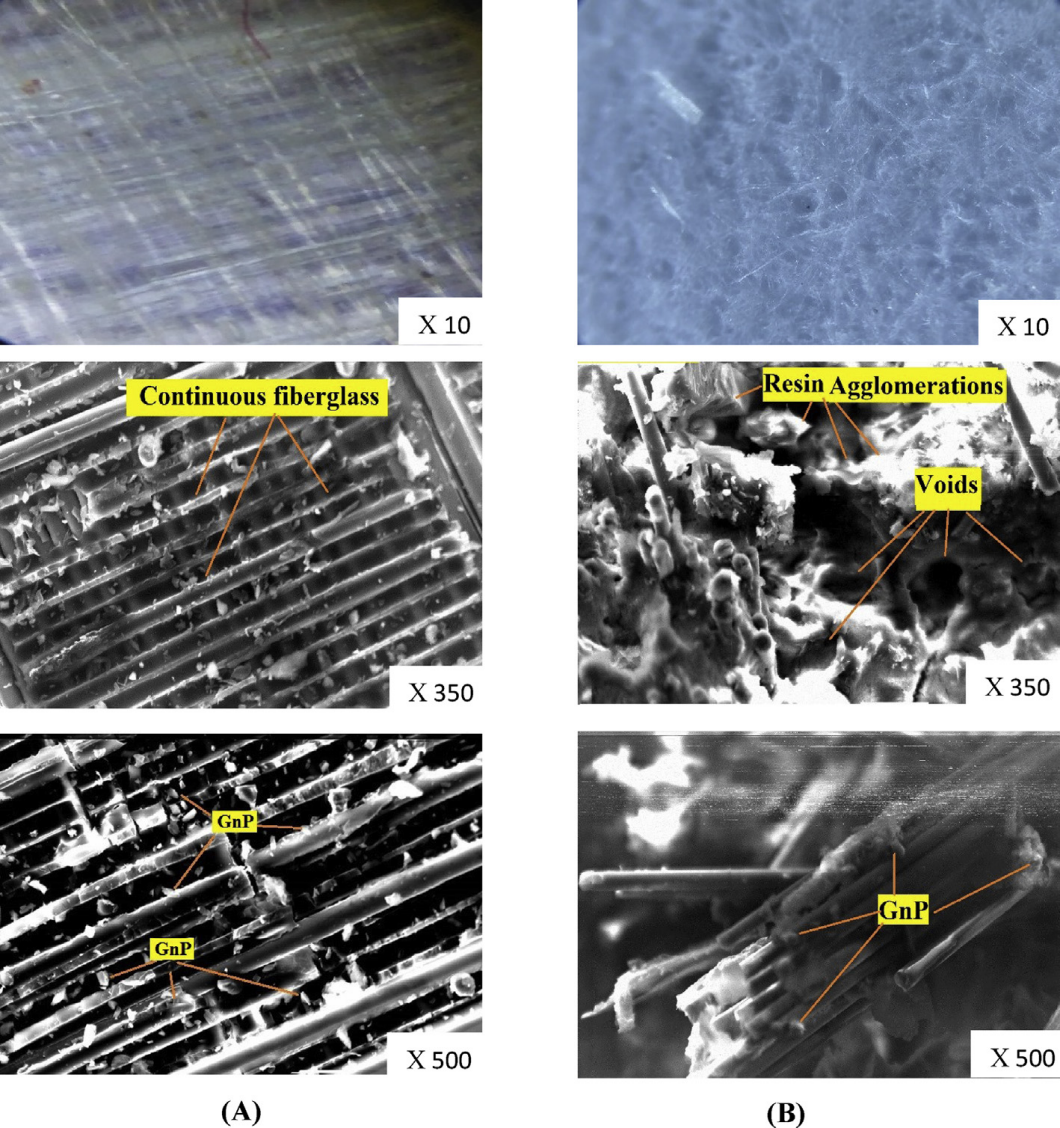
Laminte composite microstructure (A) Continuous (woven roving) fiberglass/0.5 wt% GnP.(B) Random discontinuous fiberglass/0.5 wt% GnP.

Figure 4 shows the scanning electron and microscope photograph of upper surface for laminate composite microstructure for the two types of fiberglass. It can be observed that polyurethane resin penetrated inside woven roving and no agglomeration or voids appeared in the woven roving surface (Figure 4A), on the other hand, polyurethane resin shows the bad distribution in the surface of the random discontinuous fiber and many voids and resin agglomerations appeared (Figure 4B). So, it can be concluded that woven roving samples show better distribution for the matrix compared with random discontinuous fiberglass samples. According to SEM analysis GnP, are excellent distributed over the surface of the laminate composite in the continuous fiberglass and penetrate between fibers and resin in the random fiberglass. This is due to the nature of continuous fiberglass is obstructing the Nanoplatelets from penetration between the fibers, on the other hand, the Nanoplatelets can move easily between the fibers in the random fiberglass [23].

### 3.2 Density test

**Figure 5:**
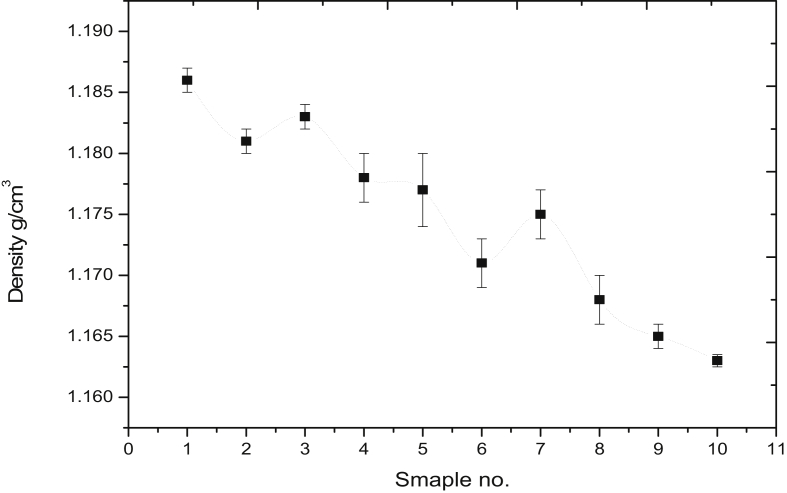
Density of the composite's samples.

Figure 5 shows the density of each sample, it is clear from the Figure the effect of the type of fiberglass and Nano filler content on the composite density [24, 25]. The density of laminate composite mainly depends upon the fraction of filler content and type of fiber. The continuous fibers samples show high density compared with random fibers samples. Increasing in the density for continuous fibers could be attributed to the spaces between the fibers layers which lead to increase the mass per unit area in continuous fibers [26]. It is clear from the Figure that the density of laminate composite decrease with the increase of the fraction of Graphene Nanoplatelets. The density of composites reinforced with 0.5 wt% of GnP is much lower than that reinforced with 0.1 wt% of GnP. This is due to that particle with high densities have high masses compared to te particles with the same size but with lower density. Since the the GnP have a small density compared with the density of fiberglass and epoxy, this lead to decrease in the density with the increase in the GnP content [27].

### 3.3 Hardness measurements

**Figure 6:**
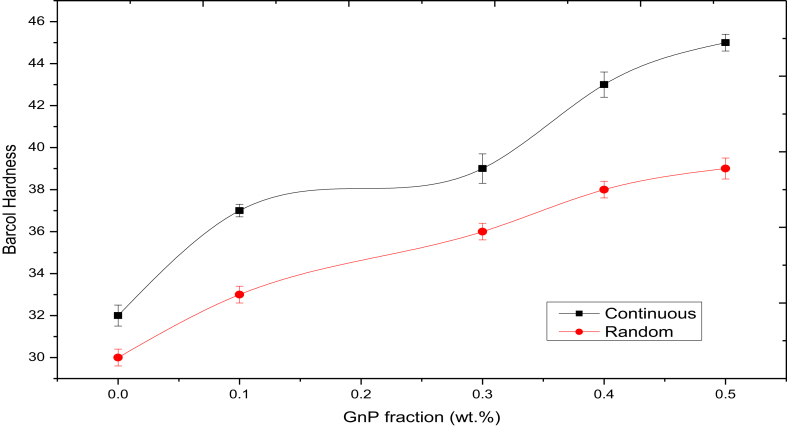
Barcol hardness for composites samples.

The testing results of Barcol hardness of the laminate composite reinforced with different fractions of GnP are exhibited in Figure 6, the results is an average of three readings. As illustrated in the Figure the Barcol hardness of the laminate composite samples is much higher after the addition GnP reinforcements when compared with that of the unreinforced samples (30 Barcol hardness for the random sample and 32 Barcol hardness for continuous fiber sample). The intensive resistance of the reinforcement laminate composite decreases the trend of the plastic deformation, which will increase the Barcol hardness of the composites [28]. The Figure reveals also, that samples with a continuous fiber (Woven Roving) show higher values compared with random fiber samples. Maximum and minimum Barcol hardness values of the composite samples were found to be 45 and 33, respectively. This decrease in the Barcol hardness values possibly resulted from voids and air bubbles that enter the random fiber samples during the preparation process [29]. In continues fiber glass the woven structure produces pockets from resin in the cross-over points which play a significnt role in enhancing the hardness values. On the other hand, in random fibers, there is a bad distribution for fibers inside the resin. This bad distribution could result from the spaces between fibers itself and inclusions resulted during resin drying leads to reduce the hardness of the composite [30].

### 3.4 Impact strength

**Figure 7:**
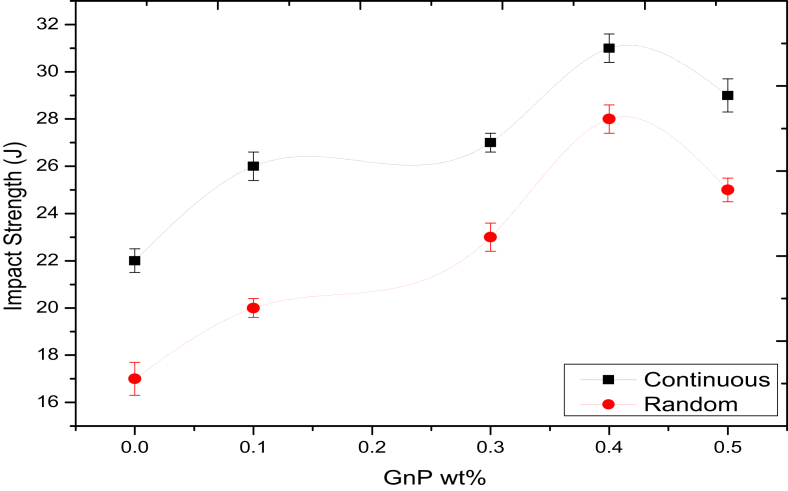
Results of Charpy impact test.

The relationship between the sample and absorbing energy is shown in Figure 7. It is clear from the results that fractions of GnP and fiberglass type had a significant effect on the absorbing energy of the composite samples. The figure shows also that absorbing energy in continuous fiberglass enhanced by 25.78, 26.74, 30.73 and 28.69 % with including 0.1, 0.3, 0.4 and 0.5 wt% GnP, respectively. The absorbing energy in random fiberglass have the same behavior, it is enhanced 19.83, 22.8, 27.77 and 24.72% with including 0.1, 0.3, 0.4 and 0.5 wt% GnP, respectively.

It is concluded that impact strength increases for both types of fiberglass from 0 to 0.4 wt% and further decreases for 0.5 wt.% GnP filler content, and samples with a continuous fiber (Woven Roving) show higher absorbing energy compared with random fiber samples. The increase in impact strength is due to that capability of absorbing energy in composites is depending on the properties of the constituents and on the strength of a bond between the fiber, resin and GnP filler [31, 32]. Because of the weak bond between the fibers and resin in random fiberglass, it showed lower absorbing energy compared with continuous fiberglass. Also the enhancing in impact strength may be due to the possibility of having a more uniform distribution of fiberglass in woven structure comparing with random structure [29] Which reduces the effect of resin pockets also the intertwined nature of woven structure helps in dispersing the effective load which increases the amount of absorbing energy. In random fiber samples, the weak bonds between the fibers and matrix (polyurethane resin) can cause propagation for the cracks through the composites, thus absorbing much less energy in the random fiber samples.

### 3.5 Thermal properties

**Figure 8:**
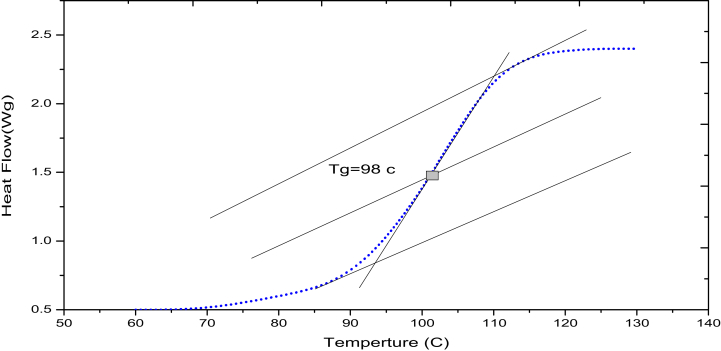
DSC data of pure polystyrene.

**Figure 9:**
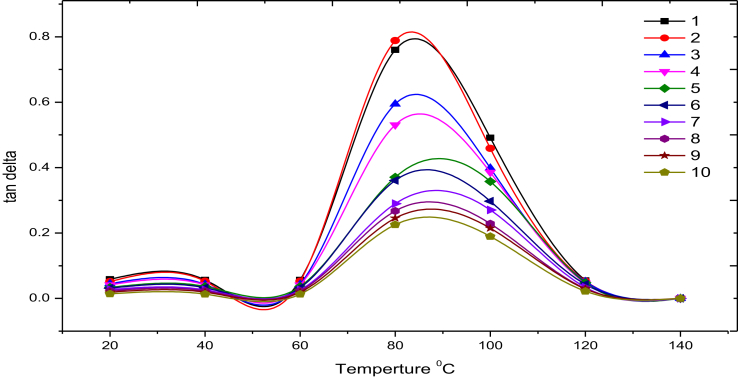
Loss tangent curves for all samples.

Based on the DSC results in Figure 8, the glass transition temperature for pure polyurethane resin is 98 °C. Figure 9 shows the effects of fractions of GnP and type of fiberglass on the values of tg delta, it can be observed that the major changes in tg delta when the composites pass the glassy to a rubbery state. In addition, the figure shows that the tg delta values decreases as the wt% of GnP are increased in the samples. This is due to that relaxation capacity of polymer chain segments decreases under the effect of the polymer-nano filler interaction [33].

**Figure 10:**
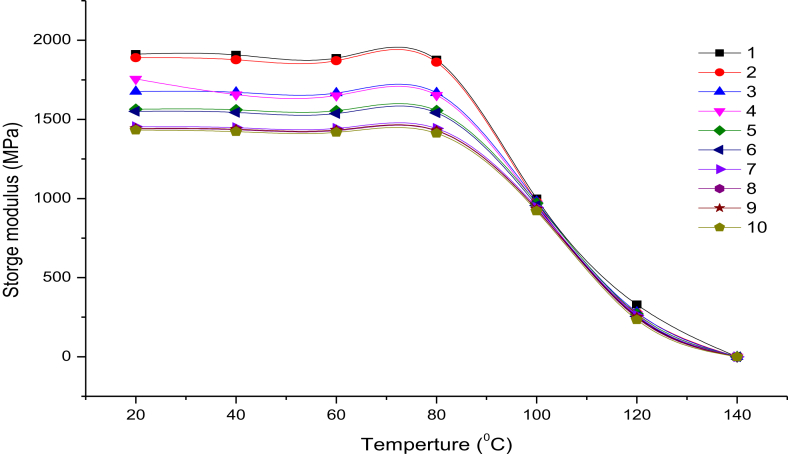
Storage modulus curves for all samples.

Figure 10 shows storage modulus curves for all samples, it can be observed that woven samples have maximum storage modulus such as the storage modulus of sample 1 at 40 C is 1892 MPa, while the storage modulus of sample 2 at 40 C is 1811 MPa. The woven structure enhances adhesion characteristics with polyurethane resin, which leads to prevent the formation of voids at the fiber matrix-reinforced interface [34, 35, 36, 37, 38].

### 3.6 Mechanical properties

#### 3.6.1 Tensile strength

**Figure 11:**
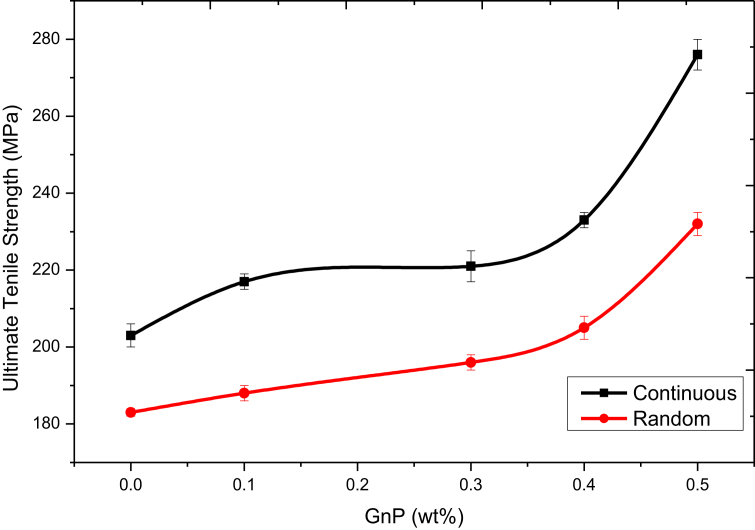
Tensile test results for random and continuous fiber samples.

In this research, the mechanical properties were studied to identify the behavior of the composite at different types of fiberglass and different wt.% of GnP. To study how the composites, deform or break as a function with applied load, ultimate tensile strength values of the different composite samples are measured and represented in Figure 11. Note that, increases by approximately 78% in continuous and random samples 82% when GnP are used as reinforced when compared to not adding them at all, and once again, the ultimate tensile strength is consistently higher in the continuous fiber samples.

Maximum tensile strength found in sample 9 was 312 MPa, hence when the tensile strength of sample 7 is compared with other samples 1,2,3,4,5,6,8 and 10 the strength of composite 9 is increased by 68%, 58%,75%,60%,81%,62%, 92%,66% and 74% respectively. The improvement in the tensile strength is due to the woven structure act as an obstruction in the stress transfer from point to another [39] and due to the higher storage modulus improves material mechanical strength as a result of the reduction in polymeric chain movement under the stiffening effect [40, 41]. The insufficient bonding between fiberglass and polyurethane resin in the random type leads to reduce the composite tensile strength. The fiber arrangement in the loading direction is an effective parameter in increasing the value of composite tensile strength [42]. The increase in the void content and agglomeration on the random fiber arrangement also effects the tensile strength [43]. According to Figure 11d, 0.5 wt.% of GnP continuous fiber has the highest tensile strength. This could be because of excellent GnP distribution. When nano plates are added, the applied force to laminate composite was easily transferred to fiber [44], so the GNP containing samples could show more tensile strength than pure laminate composite, tensile strength of 0.5 wt.% of GnP continuous fiber and 0.5 wt.% of GnP random fiber showed approximately 36 and 27 higher than that of pure laminate composite, respectively.

**Figure 12:**
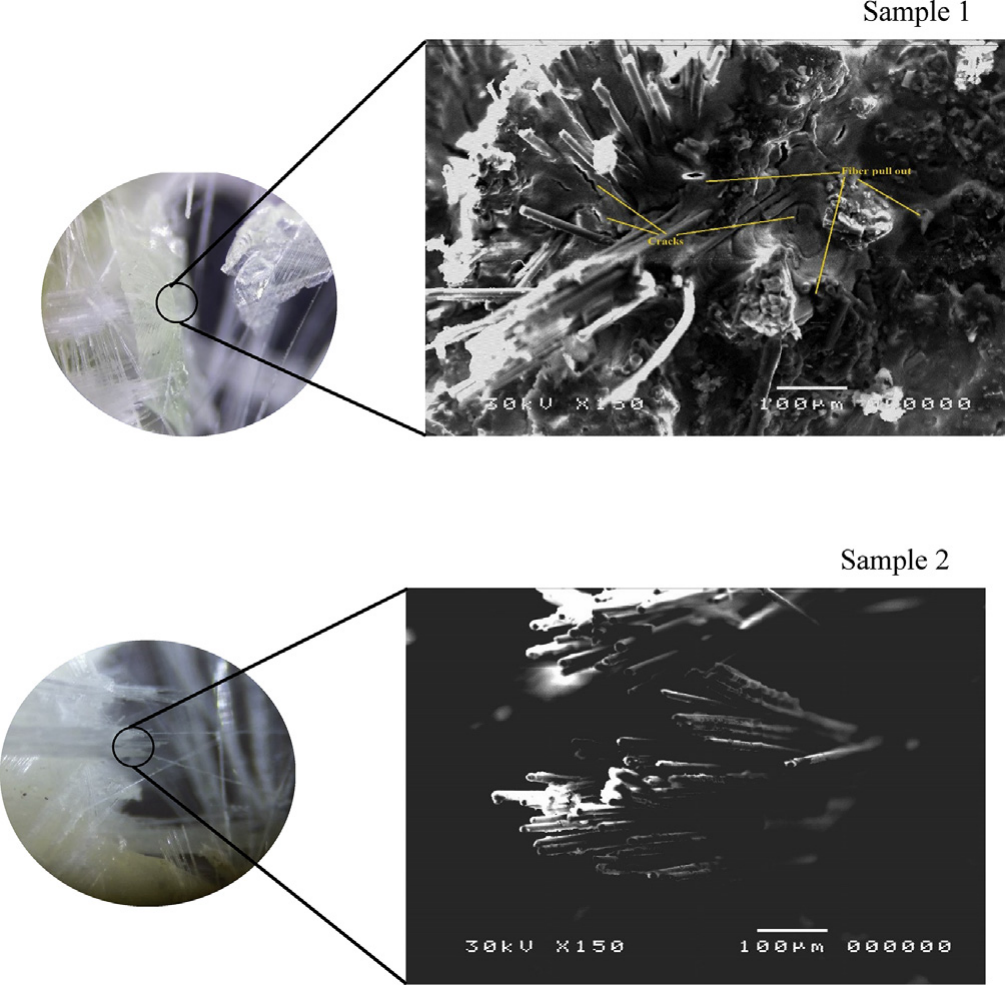
Scanning electron micrographs of tensile fracture surfaces of sample 1,2.

**Figure 13:**
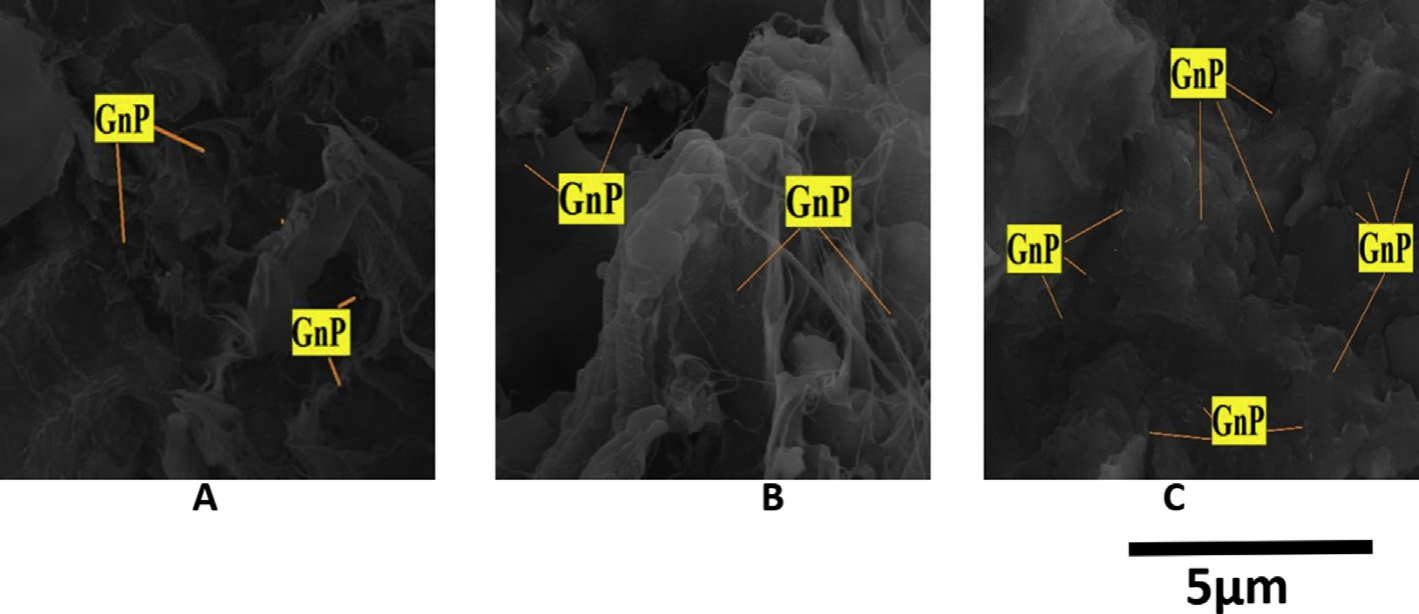
Scanning electron micrographs of tensile fracture surfaces of (A) GnP 0.1 wt%, (B) GnP 0.3 wt%, and (C) GnP 0.5 wt%.

Figure 12 is Scanning Electron Micrographs of tensile fracture surfaces of samples 1 and 2. It is clearly seen from figures that the region between neighboring plies were rich with resin and there is no effect for the GnP contents on the thickness of the resin rich region. On the other hand the the resin rich regions were thicker in the random fiber samples compared with continuous fiber samples. The figures also reveal, that the surface of the fracture runs in the weak area parallel to the fiber axis. Pull out fibers phenomena appears clearly in the random fiberglass samples (Figure 12), this is due to weak bond between the matrix and fibers. Also, it is clearly seen that the continues fiberglass shows excellent retention of polyurethane resin the broken area. On the other hand, the random fiber shows holes, voids, and uncoated fibers. Thus, the indication of better fiber/matrix bonding in the case of continuous fiber is further supported by the (SEM) study. Figure 13 shows the fracture surface of continuous fiberglass with GnP contents of 0.1, 0.3, and 0.5 wt%. The Nanoplates were in a random distribution, they were distinguished as irregular shapes and no sign of agglomeration of GnP was detected. The figures show also an excellent dispersion of GnP at the interface between the matrix and Nanoplates. Excellent dispersion of GnP indicates that improvement in the strength was achieved.

## 4 Conclusion

Laminate composites based on glass fibers with different fiber types were successfully fabricated. Analysis of their mechanical properties under an applied load, in particular, their tensile strength relative to fiber types and dispersed GnP wt%, has led to the following conclusions:

1. continuous fiberglass increases the tensile strength by 58%–92% over random fiber at different content of GnP.
2. The dispersion of 0.5 wt% of GnP producing maximum tensile strength of 312 MPa in continuous fiberglass.

The analysis of physical and thermal properties for laminate composites has led to the following conclusions.

1. Continuous fiber (Woven Roving) show higher Barcol hardness values compared with random fiber samples.
2. The density of laminate composite mainly depends upon the fraction of filler content and type of fiber.
3. The continuous fibers samples show high density compared with random fibers samples.
4. Impact strength increases for both types of fiberglass from 0 to 0.4 wt% and further decreases for 0.5 wt.% GnP filler content.
5. Woven samples have maximum storage modulus and the tg delta values decrease as the wt% of GnP is increased in both types of fiberglass.

## Declarations

### Author contribution statement

Amal Nassar: Conceived and designed the experiments; Performed the experiments; Analyzed and interpreted the data; Contributed reagents, materials, analysis tools or data; Wrote the paper.

Eman Nassar: Conceived and designed the experiments; Performed the experiments; Analyzed and interpreted the data; Contributed reagents, materials, analysis tools or data; Wrote the paper.

### Funding statement

This research did not receive any specific grant from funding agencies in the public, commercial, or not-for-profit sectors.

### Competing interest statement

The authors declare no conflict of interest.

### Additional information

No additional information is available for this paper.

## References

[1] Mohammed L., Ansari M.N.M., Pua G., Jawaid M., Islam M.S. (2015 Oct 1). A review on natural fiber reinforced polymer composite and its applications. Int. J. Polym. Sci..

[2] Neşer G. (2017 Jan 1). Polymer based composites in marine use: history and future trends. Procedia Eng.

[3] Ku H., Wang H., Pattarachaiyakoop N., Trada M. (2011 Jun 1). A review on the tensile properties of natural fiber reinforced polymer composites. Compos. B Eng..

[4] Ahmad Ishak, Azizah Baharum I.A. (2006). Composites, effect of extrusion rate and fiber loading on mechanical properties of twaron fiber-thermoplastic natural rubber (TPNR). J. Reinf. Plast. Compos..

[5] Zaini Nurul Aizan Mohd, Ismail Hanaf, Rusli Arjulizan (2018). Tensile, thermal, fammability and morphological properties of sepiolite flled ethylene propylene diene monomer (EDPM) rubber composites. Iran. Polym. J. (Engl. Ed.).

[6] Scaffaro R., Morreale M., Re G., La Mantia F. (2009). Effect of the processing techniques on the properties of ecocomposites based on vegetable oil-derived Mater-Bi® and wood flour. J. Appl. Polym. Sci..

[7] Sathishkumar T., Naveen J., Satheeshkumar S. (2014 Mar 9). Hybrid fiber reinforced polymer composites – a review. J. Reinf. Plast. Compos..

[8] Costa C.S.M.F., Fonseca A.C., Serra A.C., Coelho J.F.J. (2016 Apr 2). Dynamic mechanical thermal analysis of polymer composites reinforced with natural fibers. Polym. Rev..

[9] Monteiro S.N., Calado V., Rodriguez R.J.S., Margem F.M. (2012 Nov 15). Thermogravimetric behavior of natural fibers reinforced polymer composites—an overview. Mater. Sci. Eng. A.

[10] Stankovich S., Dikin D.A., Dommett G.H.B., Kohlhaas K.M., Zimney E.J., Stach E.A. (2006). Graphene-based composite materials. Nature.

[11] Park S., Ruoff R.S. (2009 Mar 29). Chemical methods for the production of graphenes. Nat. Nanotechnol..

[12] Wang F., Drzal L.T., Qin Y., Huang Z. (2015). Mechanical properties and thermal conductivity of graphene nanoplatelet/epoxy composites. J. Mater. Sci..

[13] Liang J., Xu Y., Huang Y., Zhang L., Wang Y., Ma Y. (2009 Jun 4). Infrared-triggered actuators from graphene-based nanocomposites. J. Phys. Chem. C.

[14] Ferry J.D. (1980). Viscoelastic Properties of Polymers. https://www.wiley.com/en-eg/Viscoelastic%2dProperties%2dof%2dPolymers,%2d3rd%2dEdition-p-9780471048947.

[15] Milas N Golubović A. (1959). Studies in organic peroxides. XXV. Preparation, separation and identification of peroxides derived from methyl ethyl ketone and hydrogen peroxide. J. Am. Chem. Soc..

[16] Cheng F., Hu Y., Yuan J. (2014). Preparation and characterization of glass fiber-coir hybrid composites by a novel and facile Prepreg/Press process. Fibers Polym..

[17] LU Devi, Bhagawan S.S., Thomas S. (2010 Jun 1). Dynamic mechanical analysis of pineapple leaf/glass hybrid fiber reinforced polyester composites. Polym. Compos..

[18] Samal S.K., Mohanty S., Nayak S.K. (2008 Oct 27). Polypropylene—bamboo/glass fiber hybrid composites: fabrication and analysis of mechanical, morphological, thermal, and dynamic mechanical behavior. J. Reinf. Plast. Compos..

[19] Pedrazzoli D., Pegoretti A. (2014 Jul 14). Hybridization of short glass fiber polypropylene composites with nanosilica and graphite nanoplatelets. J. Reinf. Plast. Compos..

[20] Burczyrński T., Kuś W. (2008 Nov 1). Identification of material properties in multi-scale modelling. J. Phys. Conf. Ser..

[21] Kandanur S.S., Rafiee M.A., Yavari F., Schrameyer M., Yu Z.-Z., Blanchet T.A. (2012 Aug). Suppression of wear in graphene polymer composites. Carbon N Y.

[22] Chatterjee S., Nafezarefi F., Tai N.H., Schlagenhauf L., Nüesch F.A., Chu B.T.T. (2012 Dec 1). Size and synergy effects of nanofiller hybrids including graphene nanoplatelets and carbon nanotubes in mechanical properties of epoxy composites. Carbon N Y.

[23] Sathishkumar T., Satheeshkumar S., Naveen J. (2014 Jul 8). Glass fiber-reinforced polymer composites – a review. J. Reinf. Plast. Compos..

[24] Mallick P.K. (1993).

[25] Daniel Gay. Composite Materials: Design and Applications, third ed.. CRC Press; 611 p.

[26] Reddy C.V., Raju C.J.S., Babu D.P.R., Ramnarayan D.R. (2017). Study of voids effect on tensile strength of carbon fiber reinforced composites for structural applications. Int. J. Res. Appl. Sci. Eng. Technol..

[27] Yousuf S., Barringer S.A. (Dec. 2007). Modeling nonelectrostatic and electrostatic powder coating. J. Food Eng..

[28] Fu X., Hu Y., Peng G., Tao J. (2017 Nov 27). Effect of reinforcement content on the density, mechanical and tribological properties of Ti3SiC2/Al2O3 hybrid reinforced copper-matrix pantograph slide. Sci. Eng. Compos. Mater..

[29] Hasan E.H., Shokry K.M., Emam A.A. (2010). Study of impact energy and hardness on reinforced polymeric composites. MAPAN.

[30] Hull D., Clyne T.W. (1996). Introduction to Composite Materials.

[31] Kaundal R., Patnaik A., Satapathy A. (2012 Jul 13). Comparison of the mechanical and thermo-mechanical properties of unfilled and SiC filled short glass polyester composites. Siliconindia.

[32] Agarwal G., Patnaik A., Sharma R. (2013). Thermo-mechanical properties of silicon carbide filled chopped glass fiber reinforced epoxy composites. Int J Adv Struct Eng.

[33] Robertson C G., Lin C J., Rackaitis M., Roland C M. (2008 Feb 28). Influence of particle size and Polymer−Filler coupling on viscoelastic glass transition of particle-reinforced polymers. Macromolecules.

[34] Worzakowska Marta (2015). Thermal and mechanical properties of polystyrene modified with esters derivatives of 3-phenylprop-2-en-1-ol. J. Therm. Anal. Calorim..

[35] Grassie N., Murry E.J.H.P. (1984). The thermal degradation of poly(-(D)-b-hydroxybutyric acid): part 2—changes in molecular weight. Polym. Degrad. Stab..

[36] Rana A.K., Mitra B.C.B.A. (1999). Short jute fiber-reinforced polypropylene composites: dynamic mechanical study. J. Appl. Polym. Sci..

[37] Nair K.C.M., Thomas S.G.G. (2001). Thermal and dynamic mechanical analysis of polystyrene composites reinforced with short sisal fibres. Compos. Sci. Technol..

[38] Karmarkar Ajay, Chauhan S.S., Jayant M., Modak M.C. (2007). Mechanical properties of wood–fiber reinforced polypropylene composites: effect of a novel compatibilizer with isocyanate functional group. Compos Part A Appl Sci Manuf.

[39] Amal Nassar E.N. (2014). Thermo and mechanical properties of fine silicon carbide/chopped carbon fiber reinforced epoxy composites. Univers J Mech Eng.

[40] Gupta1 R.K., R Hashmi KKP S.A., Srivastava A.K. (2019). Development of graphene nanoplatelets reinforced shape memory polyurethane and their DMA studies. Appl Innov Res.

[41] Jesuarockiam N., Jawaid M., Zainudin S.E., Thariq Hameed Sultan M., Yahaya R. (2019).

[42] Ockenden H.M., Welch G.A. (1955). The prepration and properties of some plutonium compounds. Part I. Plutonium hydride. J. Chem. Soc..

[43] Divakara Rao P., Udaya Kiran KEP C. (2017). Effect of fiber loading and void content on tensile properties of keratin based randomly oriented human hair fiber composites. Int. J. Compos. Mater..

[44] Yavuz Ünal H., Öner G., Pekbey Y. (2017). Comparison of the experimental mechanical properties and DMA measurement of nanoclay hybrid composites. Eur Mech Sci.

